# A Scoping Analysis of Cathelicidin in Response to Organic Dust Exposure and Related Chronic Lung Illnesses

**DOI:** 10.3390/ijms23168847

**Published:** 2022-08-09

**Authors:** Marcin Golec, Marta Kinga Lemieszek, Jacek Dutkiewicz, Janusz Milanowski, Sandra Barteit

**Affiliations:** 1 Heidelberg Institute of Global Health (HIGH), Faculty of Medicine and University Hospital, Heidelberg University, 69117 Heidelberg, Germany; 2 Department of Medical Biology, Institute of Rural Health, Jaczewskiego 2, 20-090 Lublin, Poland; 3 Department of Biological Health Hazards and Parasitology, Institute of Rural Health, Jaczewskiego 2, 20-090 Lublin, Poland; 4 Department of Pneumonology, Oncology and Allergology, Medical University of Lublin, 20-059 Lublin, Poland

**Keywords:** cathelicidin, LL-37, CRAMP, lung diseases, pulmonary diseases, organic dust

## Abstract

Over two billion people worldwide are exposed to organic dust, which can cause respiratory disorders. The discovery of the cathelicidin peptide provides novel insights into the lung’s response to organic dust; however, its role in the lung’s response to organic dust exposure and chronic lung diseases remains limited. We conducted a scoping review to map the current evidence on the role of cathelicidin LL-37/CRAMP in response to organic dust exposure and related chronic lung diseases: hypersensitivity pneumonitis (HP), chronic obstructive pulmonary disease (COPD) and asthma. We included a total of n = 53 peer-reviewed articles in this review, following the process of (i) a preliminary screening; (ii) a systematic MEDLINE/PubMed database search; (iii) title, abstract and full-text screening; (iv) data extraction and charting. Cathelicidin levels were shown to be altered in all clinical settings investigated; its pleiotropic function was confirmed. It was found that cathelicidin contributes to maintaining homeostasis and participates in lung injury response and repair, in addition to exerting a positive effect against microbial load and infections. In addition, LL-37 was found to sustain continuous inflammation, increase mucus formation and inhibit microorganisms and corticosteroids. In addition, studies investigated cathelicidin as a treatment modality, such as cathelicidin inhalation in experimental HP, which had positive effects. However, the primary focus of the included articles was on LL-37’s antibacterial effect, leading to the conclusion that the beneficial LL-37 activity has not been adequately examined and that further research is required.

## 1. Introduction

### 1.1. Rationale

Worldwide, over two billion people who work in agriculture and related industries are chronically exposed to organic dust [1,2]. This exposure may cause, trigger or deteriorate the progression of respiratory disorders such as hypersensitivity pneumonitis (HP), chronic obstructive pulmonary disease (COPD) and asthma [3]. Globally, more than 500 million people are affected by these diseases [4]. Organic dust contains several microbial components, such as Gram-negative bacteria and their major outer membrane component, endotoxin (lipopolysaccharide, LPS) [5]. Endotoxin/LPS is a common component of inhalable organic dusts and has been linked in numerous studies as a factor contributing to the etiopathogenesis of COPD [6,7,8]. LPS stimulates a vigorous response of the human organism’s immune system and causes injury to the respiratory epithelia [9,10,11]. It also provokes the expression of cathelicidin LL-37 peptide, which in turn is a potent LPS-neutralizing factor in the respiratory tract [12,13,14,15].

Hypersensitivity pneumonitis (HP) is an interstitial lung disease induced by chronic exposure to organic dust containing particles of plants, animals, fungi, or bacteria. HP is a complex clinical condition involving a cascade of immune reactions triggered and sustained by lung injury due to repeated inhalations of fine organic particles inhaled into the airways [16]. The main mechanisms of HP development are (i) chronic inflammation and (ii) pathology of repair of the damaged pulmonary epithelium. Hence, HP may be described as a pathology in which loss of lung function occurs due to fibrotic reaction in a highly inflammatory condition induced by chronic exposure to organic dust.

COPD develops due to a complex interplay between (i) host organism susceptibility, involving genetic background or respiratory tract hyper-responsiveness and (ii) long-term cumulative exposure to noxious gases and particles. The latter one traditionally meant mostly tobacco smoking; however, the latest findings and epidemiological trends indicate a growing role of air pollution: indoor and outdoor, and particularly linked to occupational exposures. Among these, a factor becoming increasingly more relevant is exposure to organic dust including organic volatile compounds and microbial load [17,18]. Furthermore, the role of microbial pulmonary infections in COPD exacerbations has been shown [19]. Pulmonary microbial infections also contribute to the course of the disease [19]. COPD is characterized by chronic inflammation sustained by the presence of eosinophils and neutrophils. Certain chemoattractants, such as IL-8, move leukocytes to the lungs in response to environmental stimuli such as cigarette smoke and microbial products, which are also components of organic dust. COPD, especially in its advanced stages, is characterized by changes in pulmonary architecture, most notably small airway remodelling with increased wall thickness due to excessive deposition of collagen. Epithelial–mesenchymal transition (EMT) is another phenomenon that contributes to the remodelling of airways in COPD.

Exposure to organic dust has also been widely investigated as a potential risk factor for asthma. According to a recent meta-analysis conducted recently by Zhang et al., organic dust exposure is a risk factor for asthma [20]. The risk of developing asthma depends on the interplay of genetic factors, gender, atopic predisposition, type of organic dust exposure, pesticide exposure, and magnitude and duration of exposure. Exposure to organic dust has been suggested as an independent risk factor for the development of asthma [21]; however, particular mechanisms are still unclear due to the complexity of the issue.

Recent advances in biomedical sciences have provided new insights into critical aspects of the response of the respiratory system to organic dust exposure, as well as the development of the aforementioned pulmonary diseases [22,23,24]. Our understanding of these processes was significantly improved due to the discovery of the LL-37 peptide, the only member of the antimicrobial peptides’ family of cathelicidins in humans [25] (see Supplementary Material S1 (Box S1) for details on cathelicidin role in the respiratory system). Following its discovery in humans in 1994, its potential for use in both pulmonary diseases [26] as well as the respiratory systems’ response to organic dust exposure was identified [25].

Moreover, findings from research into the role of cathelicidin in lung tissue response to organic dust exposure and in the aforementioned lung diseases are not limited to these specific clinical conditions but allow for tracking of the response of the pulmonary compartment to: (i) diversified microbial load, (ii) chronic inflammatory processes, and (iii) repeated lung injury and related pathologies of tissue repair [27].

### 1.2. Objectives

Numerous experimental studies have revealed that LL-37 cathelicidin, besides its core antimicrobial characteristics, is capable of exerting a wide, pleiotropic and versatile range of actions in various processes, including inflammation and tissue repair [25]. Due to the pleiotropic character of the peptide [28], studies addressing the role of cathelicidin in clinical settings have an important role in investigating cathelicidin contribution to the progression of certain diseases. A range of studies have focused on the role of LL-37/CRAMP (mouse homolog for human LL-37) in clinical settings/animal models which may contribute to a better understanding of the role of cathelicidin in these diseases. In addition, reviews investigating the role of LL-37 in the aforementioned clinical conditions were conducted. Four identified reviews addressed the role of vitamin D in selected respiratory diseases and in those only indirectly addressing cathelicidin [29,30,31,32]. One review mostly addressed the in vitro demonstrated properties of LL-37 and its plausible role in respiratory diseases based on the scientific knowledge in 2007 (Golec). Two other reviews discussed the topic of cathelicidin while discussing pathways to combat chronic inflammation in COPD [33] and analysing antiviral therapeutic approaches for human rhinovirus infections [34]. However, despite the growing interest in the role of LL-37 in the response of lung tissue to organic dust exposure and in chronic pulmonary diseases, no comprehensive review has been published to the best of our knowledge.

With this scoping review, we aim to address this limitation by mapping relevant peer-reviewed literature on LL-37 and its role in the response of lung tissue to organic dust exposure and related pulmonary diseases.

Specifically, the scoping review sought to evaluate if cathelicidin LL-37 (or its murine equivalent, CRAMP, in case of animal models) plays a role in the response to organic dust exposure and related chronic lung diseases: HP, COPD and asthma.

## 2. Results

**Figure 1:**
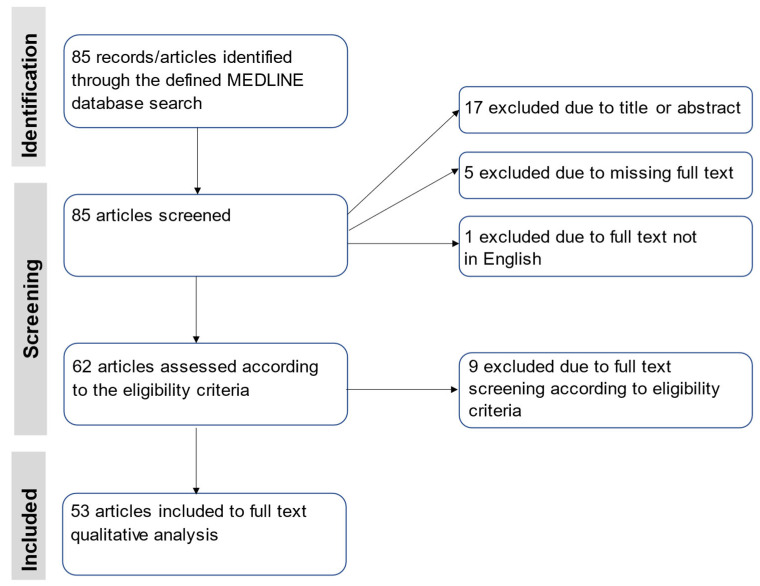
Preferred Reporting Items for Systematic Reviews and Meta-Analyses (PRISMA) flow diagram showing search process overview.

**Figure 2:**
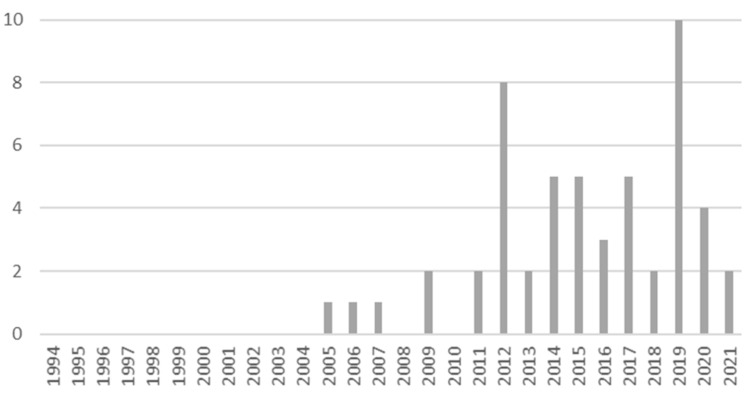
Annual distribution of published articles included in the qualitative analysis (n = 53).

We retrieved 85 articles from the MEDLINE/PubMed database, of which 53 were included in our scoping review (see Figure 1 for the PRISMA flow diagram; the full list of included articles is available in Supplementary Material S5 (Table S4)). A total of 86% (n = 46) articles were published in the last decade (2012–2021) (see Figure 2 for details). Clinical studies accounted for over half of all publications included in this review (n = 26, 49.1%), with a large number of cross-sectional studies (n = 16, 30%), followed by cohort studies (n = 10, 19%). The great majority of clinical studies (n = 17, accounting for more than 65 percent of all clinical studies) had a sample size of less than 100 people.

In vitro studies and animal models accounted for 20% (n = 11) and 17% (n = 9) of studies respectively, with seven articles representing reviews (13%). The majority of included articles originated from Europe (n = 29, 54%), followed by Asia (n = 13, 24%) and North America (n = 9, 17%). Table 1 shows the detailed characteristics of studies included in this scoping review.

In the following, we present our results in accordance with the research questions, focusing on the clinical conditions of exposure to organic dust, hypersensitivity pneumonitis (HP), COPD and asthma.

### 2.1. Exposure to Organic Dust

Six papers (11.3%) addressed the role of cathelicidin in exposure to organic dust, including three animal model studies (5.7%), two clinical investigations (4%) and one review (1.6%).

Cathelicidin levels were found to be increased in the pulmonary compartments of COPD patients following exposure to LPS, the component of the wall of Gram-negative bacteria as well as a potent constituent of organic dust [35]. Cathelicidin concentration was found elevated in farmers occupationally exposed to organic dust, compared to healthy urban dwellers [12]. It was assumed that the above-mentioned rise in LL-37 was related to the observed activities of cathelicidin: inactivation of LPS, removal of bacteria from airways, and acceleration of tissue damage repair in pulmonary epithelia caused by exposure to organic dust [12].

### 2.2. Hypersensitivity Pneumonitis

Three papers (one review [25] and two experimental studies involving animal models [36,37], 5.7%) were identified as addressing the role of cathelicidin in HP. Golec et al. (2015) observed significant changes in the CRAMP level in the pulmonary compartment during experimental HP, indicating a role for cathelicidin in HP. Cathelicidin decreased during the course of HP, which was associated with fibrotic lung tissue lesions [37]. The identified decrease in the factor enhancing epithelial repair may result in impaired lung tissue repair and a shift towards fibrosis [37]. Another review [25] postulated the plausible role of cathelicidin in HP based on the analysis of the peptide properties demonstrated in in vitro experiments. An experimental study underlined the usefulness of CRAMP in the treatment of pulmonary fibrosis assessed in a murine model of hypersensitivity pneumonitis [36]. In particular, it was demonstrated that CRAMP attenuated the immune response induced by chronic exposure of mice to potent constituents of organic dust (saline extract of Pantoea agglomerans) and inhibited the deposition of hydroxyproline and collagen in the lung tissue of mice treated with bacteria extract [36]. The study found that the beneficial effect of CRAMP on HP treatment was associated with restoring a balance in quantity of immune cells, cytokine production and synthesis of extracellular matrix components. Thus, it suggested that cathelicidin may be useful in preventing lung fibrosis.

Despite the study’s finding that cathelicidin was unable of completely reverse pathological changes, this suggests that the fibrotic process is the result of a complex interplay between a number of components, or that cathelicidin is merely one among a number of significant players.

### 2.3. COPD

The role of cathelicidin in COPD was addressed in the majority of included articles, 34 papers (64%). These included 19 clinical investigations (56% of all papers discussing cathelicidin activity in COPD), nine in vitro studies (26%), two animal model studies (6%) and four reviews (12%) (see Supplementary Material S5 (Table S4) for an overview). In the following sections, we focus on the properties of cathelicidin in COPD and describe the included studies.

#### 2.3.1. Changes in Cathelicidin Level in COPD

Changes in cathelicidin levels in COPD were documented by 84% (16/19) of the clinical studies included in the review and by all clinical studies that addressed the topic (see Supplementary Material S5 (Table S4) for details). The findings of twelve clinical studies (12/19, 75%; 63% of all clinical studies included in the review) revealed a rise in LL-37 levels during the course of COPD in the pulmonary compartment (n = 10) or in the plasma (n = 2) (see Supplementary Material S5 (Table S4) for details). Furthermore, three studies (3/19, 18%) showed significant differences between early and advanced COPD stages according to the internationally recognized Global Initiative for Chronic Obstructive Lung Disease (GOLD) classification [38]. Namely, Golec et al. observed significantly higher LL-37 levels in material from the pulmonary compartment (ELF and BALF) in early stages of COPD (GOLD I–II) compared to advanced stages (GOLD III–IV) [39]. Uysal et al. (2019), in their study exceeding 200 participants, noted in plasma significantly lower LL-37 level in advanced COPD (GOLD IV) compared to earlier stages (GOLD I–III) [40]. Significant differences between early and advanced COPD phases were also found in sputum by Jiang et al.; however, in this case, cathelicidin concentration was increased in GOLD III–IV compared to earlier stages [35]. These results suggest profound changes in non-specific immunity during progression of COPD where a period of enhanced activity of antimicrobial defence as a result of recurrent bacterial contamination of the pulmonary compartment may be followed by another stage characterized by reduced non-specific antimicrobial activity [39].

Furthermore, Persson et al. in their study involving >200 participants, described elevated levels of LL-37 as a part of the antimicrobial defence mechanisms in the pulmonary compartment (sputum) during COPD exacerbations, caused by bacterial infections [39]. The level of LL-37 in plasma of individuals with high risk of COPD exacerbations was decreased, according to Yang et al. [41]. According to Yang et al., low plasma levels of LL-37 and 25 (OH)D might be predictors of exacerbation risk in COPD patients; however, further research is necessary to confirm this inference and to explain the mechanism behind this phenomenon [41].

#### 2.3.2. Cathelicidin Level Correlates with Lung Function

Two clinical studies (n = 2/19, 11% of all clinical studies included in the review) reported the negative correlation of cathelicidin in plasma [42] and sputum [43] with lung function. Both sputum and plasma cathelicidin levels were found to be negatively correlated with lung function [42,43]. Both findings, conducted by Burkes at al. and Wright et al., have to be seen as especially thorough, as they derive from clinical studies with a high number of participants, namely exceeding 500 [42] and 100 individuals [43]. Furthermore, one study conducted by Jiang et al. (5%, n = 19) noted that increased induced sputum LL-37 levels in COPD patients were also associated with airflow limitation [35]. Jiang et al. hypothesized that the reason behind these correlations might be the contribution of LL-37 to the progression of COPD. The latter included causing epithelial apoptosis by LL-37, as epithelial cell apoptosis is as one of important mechanisms of pulmonary emphysema [35].

#### 2.3.3. Cathelicidin as an Antimicrobial Agent in Combating Infections in COPD

Three studies (n = 3/34 addressing the role of cathelicidin in COPD, 9%,) reported cathelicidin to increase defence against microbial infections that contribute to COPD exacerbation [44,45,46]. In both clinical studies and animal models, both bacterial and viral infections were associated with an increase in cathelicidin levels and an elevation of cathelicidin gene coding [44,45,46]. In the study by Tangedal et al. (2019), individuals with COPD exacerbations had considerably greater LL-37 levels in their sputum than individuals with stable COPD [47].

#### 2.3.4. Cathelicidin Contributes to COPD Development

Cathelicidin was found to contribute to processes that underpin the pathophysiology of COPD and to drive the progression of COPD. Thus, LL-37 increases persistent inflammation and sputum production and contributes to the remodelling of COPD airways.

##### Cathelicidin Sustaining Persistent Inflammation in COPD

Three studies (n = 3/34, 9%) demonstrated cathelicidin’s role in the remodelling of lung tissue in COPD. Tjabringa et al. (2006) showed in their in vitro study that LL-37 exerts chemotactic activity for neutrophils and eosinophils [48]. According to two other studies, cathelicidin appears not only to respond to microbial load but also to contribute to sustained, persistent inflammation [49,50]. Specifically, Bozinovski et al. (2014) found that the elevated levels of LL-37 in COPD make this peptide a potent factor sustaining persistent, non-resolving inflammatory factor, thereby contributing to the disease’s development [49]. The findings of Pouwels et al. [50] reinforce this conclusion by demonstrating that cathelicidin, as a powerful pro-inflammatory chemical, contributes to the development of COPD in clinical, experimental, and animal model settings.

##### Cathelicidin Contributes to Lung Tissue Remodelling in COPD

Two papers (n = 2/34, 6%) demonstrated cathelicidin’s role in the remodelling of lung tissue in COPD. Jiang et al. (2021) showed that cathelicidin induces airway EMT in the animal model of COPD [50]. Moreover, Sun et al. (2014) found that LL-37 promotes collagen production in small human lung fibroblasts in the presence of COPD causative factors, e.g., cigarette smoke [51]. The latter was shown to take place via the formyl peptide receptor-like 1 (FPRL1)-dependent extracellular signal-regulated kinase (ERK) signalling pathway. Furthermore, Sun et al. (2014) discovered a link between LL-37 expression in the epithelium and structural changes associated with small airway remodelling [51].

##### Cathelicidin Enhances Mucous Production in COPD

Two papers (n = 2/34, 6%) demonstrated that cathelicidin enhances mucous production in COPD. Zhang et al. demonstrated that LL-37 increases mucus production in COPD airways [52], which is a characteristic of COPD and contributes to the disease progression [52]. In another study, Zhang et al. confirmed this finding, as they demonstrated that cathelicidin dose-dependently induces mucin formation in airway epithelial cells via the TACE-TGF–EGFR pathway [53].

#### 2.3.5. Cathelicidin and Corticosteroids Treatment in COPD and Asthma

Three papers (n = 3/34, 9%) evaluated the interplay between cathelicidin and commonly used COPD and asthma drugs: corticosteroids [54,55,56]. Van der Berge et al. found no difference in CAMP/LL-37 gene expression between budesonide and fluticasone propionate treatment [51].

Weng et al. evaluated if cathelicidin could contribute to the overcoming of corticosteroid resistance, which can occur in COPD and asthma. In their study of a COPD animal model, they found that this may result from an increase in histone deacetylase-2 (HDAC2) activity (lower activity of HDAC2 is one of the potential causes for decreased effects of corticosteroid therapy) [55]. In a clinical setting, Singanayagam et al. (2019) found that corticosteroid treatment inhibits the activity of cathelicidin [54]; therefore, increasing the level of cathelicidin in the pulmonary compartment, either directly through extrinsic LL-37 or indirectly through vitamin D supplementation, should be considered.

### 2.4. Asthma

The role of cathelicidin in asthma was addressed by 27 studies (51%), including ten clinical investigations (19%), six in vitro studies (11.3%), five animal model studies (9.4%) and six reviews (11.3%) (see Supplementary Material S5 (Table S4) for details).

#### 2.4.1. Cathelicidin Levels Are Altered in Asthma

Six clinical studies (n = 6/10, 60%; proportion of papers addressing the role of cathelicidin in asthma included in the review) found that cathelicidin levels decreased along the course of asthma (see Supplementary Material S6 (Table S5) for details). Specifically, two studies noted significantly lower cathelicidin levels in asthma patients compared to healthy controls [43,57], and three studies (30%) found significantly decreased cathelicidin levels in asthma compared to COPD [43,57,58]. According to Huang et al. [53] and Xiao et al. [57], cathelicidin concentration in sputum may be a biomarker separating COPD from asthma. Furthermore, two studies (20%) by Arikoglu et al. (2015) and Arikoglu et al. (2017) found lower LL-37 levels in stable asthma versus asthma exacerbations [59,60].

Rhode et al. (2014) and Thijs et al. (2015) in their clinical studies (20%) observed no change in cathelicidin concentration [61,62].

#### 2.4.2. Cathelicidin Enhances Inflammation in Asthma

Two studies included in the review (n = 2/27, 7%, n = 27) seemed to suggest that LL-37 increases and sustains inflammation in the lungs over the course of asthma by acting as a chemotactic factor for eosinophils and neutrophils [48] and boosting the release of proinflammatory cytokines [63]. According to the findings of the in vitro study by Jiao et al. (2017), cathelicidin may contribute to the development of asthma or potentially trigger exacerbations [63]. Tjabringa et al. (2006) suggested that LL-37 modulates inflammation in asthma by selectively recruiting inflammatory cells as a treatment for inflammatory lung diseases such as asthma [48].

#### 2.4.3. Cathelicidin as an Antimicrobial Agent in Combating Infections in Asthma

Two studies included in the review (n = 2/27, 7%), conducted by Jiao et al. (2017) and Casanova et al. (2018), suggest that elevated levels of cathelicidin in the lungs during asthma exacerbation aid in combating microbial infections [63,64].

#### 2.4.4. Cathelicidin and Vitamin D in Asthma and COPD

Six papers (n = 6/27, 22%; of all studies addressing the role of cathelicidin in asthma included in the review) addressed the role of vitamin D in altering the course of asthma by influencing cathelicidin level.

Vitamin D stimulates the production of cathelicidin in lung tissue, and according to four review papers included in this review (n = 4/27, 15%), this is the primary reason why its deficiency affects asthma by increasing the risk of microbial infections and exacerbations [55,56,57,58]. Therefore, Székely et al. (2012) proposed using vitamin D supplementation to increase cathelicidin concentrations in the lungs in order to ameliorate the course of both asthma and COPD [58].

Greiller et al. (2019) conducted experiments as part of their vitro study to evaluate the effect of vitamin D on responses of respiratory epithelial cells to infection with rhinoviruses, a common respiratory tract pathogen [59]. Greiller et al. (2019) linked vitamin D effects to increased expression of cathelicidin [59]. However, the clinical study conducted by Arikoglu et al. (2015) only partially validated the conclusion of Greiller at al. (2019). Arikoglu et al. (2015) found a significant relationship between vitamin D insufficiency and the development of asthma exacerbations; however, this effect was independent of cathelicidin levels [60]. Arikoglu et al. (2015) suggested the need for more research to evaluate the relationship between these two molecules in the development of asthma, given their growing importance, e.g., as possible therapeutical targets [60]. In respect to cathelicidin, no clinical studies on the impact of vitamin D in COPD were found.

## 3. Discussion

The findings of the review confirmed a growing scientific interest in the role of cathelicidin in exposure to organic dust and related chronic lung diseases (HP, COPD, asthma). Eighty-six percent of the included articles were published in the last decade (2012–2021), whereas only 14% were published in years earlier to 2012 (1994–2011).

We conducted this scoping review to map the current evidence on the role of cathelicidin LL-37/CRAMP in response to organic dust exposure and related chronic lung diseases. Particularly COPD, which affects over 174 million people worldwide, is one of the most prevalent chronic respiratory diseases worldwide [65] and remains one of the major global public health challenges.

Overall, cathelicidin levels were found to be altered in both the pulmonary compartment and plasma for clinical settings considered in this review (COPD, asthma, HP, exposure to organic dust) [12,35,39,40,41,43,47,57,59,60,61,66,67]. These findings, along with the properties of the peptide in in vitro studies, suggest that cathelicidin plays a mostly beneficial role in these diseases. Recent studies suggest that LL-37 levels are elevated in COPD GOLD I and II but decrease in advanced COPD and HP stages [12,37,39,40]. This may be attributed to pathological changes in lung tissue, presumably of a fibroproliferative nature, that are not present in earlier stages of COPD. In advanced stages of COPD, decreased LL-37 levels may be particularly high risk due to an important role of the peptide in antimicrobial defence [40]. Moreover, the patterns of cathelicidin levels in asthma (decreased in a stable state) and COPD (increased in a stable state of GOLD I and II) allows us to hypothesize that LL-37 could serve as a biomarker for distinguishing between these two diseases [31,43,57,58].

We found a negative correlation between cathelicidin levels and lung function in COPD [27,37,38], as well as no correlation between LL-37 levels and lung function. Furthermore, we found that both microorganisms and corticosteroids develop mechanisms to neutralize LL-37 (citrullination), and both are commonly used in asthma, HP, and COPD and impede LL-37 activity in this manner [55,56,68,69]. Both findings suggest a possible role of the need of considering methods to increase cathelicidin levels in the pulmonary compartment as a potential therapeutic target.

The results of studies targeting the role of cathelicidin in clinical settings in COPD, asthma and HP confirmed the versatile and pleiotropic function of this peptide, which had previously been described in several in vitro studies [25]. Cathelicidin seems to aid in the regulation of homeostasis and participates in the response to injury and repair processes in the pulmonary compartment, whether caused by organic dust or lung diseases such as COPD, asthma and HP. Cathelicidin levels are elevated in COPD and asthma exacerbations, as well as lung infections caused by pathogenic bacteria and viruses, suggesting that cathelicidin has a beneficial role in these clinical setting [44,45,46,63,64]. This beneficial effect includes its antimicrobial and LPS-neutralizing activity.

However, LL-37 may potentially contribute to the development of COPD, especially in early stages of the disease, when cathelicidin concentration is elevated. Additionally, LL-37 was shown to increase mucus production [52,53] and to add to airway remodelling (collagen production, wall thickness, etc.) in COPD. In addition, the findings showed that LL-37 contributes to persistent inflammation in lung tissue in COPD [33,35,43,48,49,66] and asthma patients [48,63]. In general, the progression of COPD and asthma is driven by persistent inflammation, increased mucus production, and pathological remodelling of lung tissue.

Due to the beneficial effects of cathelicidin in the pulmonary diseases of COPD, asthma and HP, research is being conducted to determine ways to increase LL-37 levels [70]. As vitamin D has been shown to increase the expression of cathelicidin, its supplementation has been used as a relatively simple means to enhance LL-37 in the pulmonary compartment, leading to decreased risk of microbial infections and thus to exacerbation in such clinical settings such as COPD [29,32,64,71] and asthma [29,30,31,60]. Furthermore, cathelicidin inhalation was used in an experimental HP treatment, showing a number of improvements such as a potential inhibition of fibroproliferative tissue remodelling, as well as decreased hydroxyproline and collagen deposition in lung tissue [36].

Regarding HP, Lemieszek et al. (2021) investigated the role of inhaled cathelicidin in suppressing fibrotic lung tissue remodelling in experimental HP and found that cathelicidin is able to inhibit hydroxyproline and collagen deposition in the lung tissue during experimental HP. This finding shows that this peptide may be effective in preventing pulmonary fibrosis [36].

The therapeutic potential of cathelicidin for a variety of clinical diseases is emphasized by the positive effects of cathelicidin, which are consistent with other studies. In their in vitro study, Sousa et al. (2017) demonstrated the effectiveness of cathelicidin in neutralizing human rhinoviruses, a common cause of viral respiratory tract infections [72]. Vitamin D supplementation was presented as a good approach for increasing LL-37 levels [64], and its beneficial role appears to extend to rhinoviral or respiratory infections as well [73].

As a potent component of the innate/non-specific immune system, cathelicidin has been demonstrated to be a potential player a role in a range of pulmonary diseases involving inflammatory processes, recurring infections, and microbial burden. For example, in pulmonary compartments of patients with sarcoidosis, LL-37 levels were observed to be considerably higher [74], similarly for cystic fibrosis [62]. This effect seems to be related to LL-37’s role in supporting inflammatory and antimicrobial defence mechanisms.

Our scoping review discovered a significant knowledge gap regarding the role of cathelicidin in organic dust exposure and its role in HP, as well as its role in COPD or asthma in relation to organic dust exposure. Due to the current emphasis in using LL-37 as a therapeutical/diagnostic target, the widespread global exposure to organic dust, and the fact that COPD and asthma are major global public health concerns, this gap must be regarded as significant.

### Limitations

Our scoping review has a number of limitations. First, only six studies (11.3% of all identified studies) examined the role of cathelicidin in organic dust exposure, and only three studies (6%; no clinical studies) addressed the role of cathelicidin in HP. Given the paucity of research studies, it was impossible to analyse the role of cathelicidin in COPD (only one paper included; 2%) or asthma (no study) in relation to exposure to organic dust. Another limitation of the scoping review was that we solely analysed the role of cathelicidin in COPD and asthma, without analysing the interaction between organic dust exposure, COPD or asthma, and cathelicidin’s function.

In addition, no studies have been identified that examine the role of cathelicidin in diseases caused by exposure to organic dust in organs other than the lungs and respiratory tract (skin, eye, etc.). Regarding clinical trials, another limitation of the scoping review was that, in virtually all instances, they comprised only a small sample size; only one clinical study (3.8% of all clinical studies) involved more than n = 500 participants, while five studies (19.3%) only engaged between 200 and 500 participants (see Table 1 for details; see Supplemental Material S5 (Table S4) for detailed characteristics of clinical studies).

## 4. Materials and Methods

Due to the broad scope of the research question and the aim to include all types of studies, a scoping literature review seemed to be the most adequate approach to accomplish our research objectives. The stages of the review were established in accordance with the methodological framework proposed by Aksey and O’Malley [75] and modified by Levac et al. [76]: (1) formulating the research question according to the framework of Population, Intervention, Comparison, Outcomes and Study (PICOS); (2) identifying relevant studies and searching them with a MEDLINE/PubMed (pubmed.ncbi.nlm.nih.gov) database search; (3) screening of studies to filter the studies according to inclusion/exclusion criteria; (4) charting the data, including a review of the relevant papers; (5) collating and summarizing the results.

The review was reported in line with the Preferred Reporting Items for Systematic Reviews and Meta-Analysis–Scoping Review (PRISMA-ScR) framework [77] (see Supplementary Materials S2–S4 (Tables S1–S3) for the review protocol with detailed instructions, inclusion/exclusion criteria, and a data extraction form).

### 4.1. Search Strategy

We systematically searched the MEDLINE/PubMed database between 16 and 17 October 2021, using the following search terms: “asthma”, “COPD”, “hypersensitivity pneumonitis”, in conjunction with (AND) the terms “LL-37”, “cathelicidin”. The following search strings were used: (“organic dust” OR “asthma” OR “COPD” OR “hypersensitivity pneumonitis”) AND (“CRAMP” OR “cathelicidin” OR “LL-37”). Based on test searches, we identified adequate synonyms, Medical Subject heading (MeSH) phrases, and additional keywords, and altered the final search string to match the syntactic criteria of the MEDLINE/PubMed database. Furthermore, we handsearched references of relevant studies.

### 4.2. Inclusion and Exclusion Criteria

We used the PICOS framework to develop inclusion and exclusion criteria that also steered the screening process [78]. Studies were included if they evaluated cathelicidin LL-37/CRAMP in organic dust exposure or at least one of the respiratory diseases that may be caused by organic dust exposure, which are largely HP, COPD, and asthma at present. Only papers addressing the role of the LL-37/CRAMP peptide in the specified respiratory diseases, whether experimental, clinical or based on animal models, were included. No studies were excluded based on their study design. Only peer-reviewed research published after 1994, when human cathelicidin was characterized, and available in full text, were considered. Peer-reviewed papers published in languages other than English, Polish, German or French, editorials, letters to the editor, commentaries and press articles were excluded (see Annex 2 for details on the eligibility screening form).

### 4.3. Selection of Studies

The review team was composed of four researchers (MG, MKL, JD, JM) with expertise in medicine and biotechnology, working in pairs. Each group was randomly assigned 50% of the papers. Each paper was reviewed independently by two reviewers. Disagreements amongst reviewing researchers were resolved through brief discussions (see Supplementary Materials S2 and S3 (Tables S1 and S2) for details on screening).

### 4.4. Charting the Data

The data extraction form was handed to all reviewers in MS Office Excel format (see Supplementary Materials S2–S4 (Tables S1–S3)). This form included one closed and three open-ended questions to guide the data charting process. Using open-ended questions, we identified the article’s main objective and most significant results. We extracted the author, title, and year of publication as well as article domain, objectives, description of the role/behaviour of cathelicidin in a selected lung disease or in exposure to organic dust, and key results.

## 5. Conclusions

Overall, this scoping review was able to contribute to the understanding of the current state of research on the role of cathelicidin in organic dust exposure and organic dust-induced respiratory diseases. In particular, our scoping review underlines the role of cathelicidin in a range of clinical diseases, including organic dust exposure, COPD, asthma and HP. We have found an increasing number of studies addressing cathelicidin’s role in the clinical conditions of COPD, asthma and HP. Overall, this role appears to differ, depending on the stage (early/advanced) of COPD or the form (stable/exacerbation) of asthma. Furthermore, cathelicidin seems to play a pleiotropic and versatile role in these diseases, both adding to the development of these pathologies as well as exerting its beneficial role. However, except for its antimicrobial property, its beneficial role has not yet been sufficiently investigated. Cathelicidin’s wound-healing properties, its involvement in accelerating lung tissue repair in the presence of organic dust, and its effects on COPD, asthma, and HP have not been sufficiently investigated. A further important research gap identified by our scoping review is the paucity of studies analysing the effect of cathelicidin in COPD and asthma patients caused by organic dust. Potential efforts to use cathelicidin as a therapeutic agent or therapeutic target in these clinical conditions should be preceded by research into the wound-healing and tissue-repair properties of this peptide, as well as cathelicidin’s behaviour in COPD and asthma when exposed to organic dust. A recent experimental study utilising cathelicidin as an agent to inhibit fibroproliferative tissue remodelling in experimental HP showed encouraging results [36]. In addition, emphasis should be given to the suppression of cathelicidin activity by microorganisms or medications widely used to treat COPD, asthma, and HP.

## Figures and Tables

**Table 1:** Characteristics of studies included in the scoping review (n = 53).

Clinical Setting *		Study Type **	
Exposure to organic dust	6 (11.3%)	clinical study	26 (49.1%)
HP	3 (5.6%)	cohort	10 (18.9%)
COPD	34 (64.1%)	cross-sectional	16 (30.2%)
Asthma	27 (51%)	animal model	9 (17%)
		in vitro	11 (20.7%)
**Publication Years**		review	7 (13.2%)
1994–2011	6 (14%)	Peptide **	
2012–2021	46 (86%)	LL-37	45 (84.9%)
**Regions**		CRAMP	10 (18.9%)
Europe	29 (54.7%)	**Sample Size of Clinical Studies ^#^**	
Asia	13 (24.5%)	<100	17 (65.4%)
North America	9 (17.0%)	100–200	3 (11.5%)
Australia and Oceania	2 (3.8%)	200–500	5 (19.3%)
		>500	1 (3.8%)

* One paper addressed all these conditions: exposure to organic dust, HP, COPD and asthma; nine articles addressed COPD and asthma; three studies addressed exposure to organic dust and COPD; two articles addressed exposure to organic dust and HP; ** two papers addressed both LL-37 and CRAMP peptides. ^#^ m = 26.

## Data Availability

Not applicable.

## Supplementary Materials

The following supporting information can be downloaded at: https://www.mdpi.com/article/10.3390/ijms23168847/s1. References [79,80,81,82,83,84,85,86,87,88,89,90,91,92,93,94] are cited in the supplementary materials.
