# Supplementary material for: A Scoping Analysis of Cathelicidin in Response to Organic Dust Exposure and Related Chronic Lung Illnesses
Source: Int J Mol Sci. 2022 Aug 9;23(16):8847. doi: 10.3390/ijms23168847 (PMC9408003; doi:10.3390/ijms23168847)
Supplement: Supplementary file 1 [file ijms-23-08847-s001.zip › ijms-1833505-supplementary.pdf]

International Journal of
Molecular Sciences

MDPI

Supplementary Material S1

Box S1. Cathelicidin LL-37 in respiratory system.

Cathelicidin LL-37 in respiratory system

Bactenecin, derived from the Latin expression "*bacterium necare*", which means "killing bacteria", was the initial name given to the first cathelicidin peptide found in mammals about forty years ago [89, 90]. The human cathelicidin, LL-37 peptide, is the only member of the cathelicidin family that has been found in humans. Concerning animal models, the mouse cathelicidin, CRAMP (Cathelicidin Related Microbial Peptide) was considered [91]. LL-37/hCAP18 is an 18 kDa peptide encoded by the gene CAMP. LL-37/hCAP18, later called LL-37, is a small peptide of 37 amino acid residues starting with two leucine residues [92].

LL-37 is an important part of innate immunity and, as such, is produced by cells, which are exposed to the external environment, including respiratory epithelial cells [25]. Cathelicidin is secreted to the fluid layer lining airways and alveoli, where it exerts a broad antimicrobial activity. LL-37 performs its bactericidal action by electrostatic binding of its cationic molecules to the outer surface of the bacterial cell. Insertion of the peptide into the cell membrane causes cytoplasm to leak into the extracellular space resulting in the death of the bacterial cell [93].

Yet, what makes LL-37 peptide so unique is that the activity of this relatively basic and small peptide is not limited to its antimicrobial capacity but rather exerts a multitude of actions involving various cells and cytokines. This enables human cathelicidin to participate in regulating inflammation and tissue repair processes [25], enhancing angiogenesis [94], stimulating proliferation of lung epithelial cells [89], accelerating wound closure of the airway epithelium[89], and provoking cytokine release (e.g. IL-8) and cell migration [94] as well as causing cell necrosis [89].

Gram-negative bacteria create LPS (endotoxin), a heteropolymer linked with organic dust that is neutralised by human cathelicidin. Thus, LL-37 protects human organisms against endotoxic shock [15]. LL-37 binds to LPS, dissociates endotoxin aggregates and competes with LPS for the CD14 receptor binding site, thereby preventing endotoxin-dependent cytokine induction and macrophage activation [15]. Endotoxin contributes to the pathogenesis of chronic respiratory diseases caused by organic dust, including hypersensitivity pneumonitis (HP), chronic obstructive pulmonary disease (COPD) and asthma [25].

Supplementary Material S2

Table S1. Relevance screening form on basis of bibliographic data (title and abstract).

RELEVANCE SCREENING FORM ON BASIS OF BIBLIOGRAPHIC DATA (TITLE AND ABSTRACT)
**Authors, title, Year of publication, DOI/PMID, journal and other bibliographic data:**

No	Question	Options	Exclusion if	Additional notes
1	What type of source is the result?	Journal Paper (go to question 2)	Result of category “others”	
		Conference Paper(go to question 2)		
		Book Section (go to question 2)		
		Unpublished Work (go to question 2)		
		Commentary (go to question 2)		
		Editorial (go to question 2)		
		Others (e.g. press article)		
2	Is an abstract available?	Yes (go to question 3)		
		No (go to question 2a)		Please use existing links and try to find the abstract using search engines
3	It can be concluded from the title and abstract that the article deals with: i) cathelicidin in selected pulmonary diseases: COPD, asthma, HP; OR ii) cathelicidin in relation to organic dust exposure.	Yes (go to question 3)	No	
		No		

Supplementary Material S3

Table S2. Eligibility Screening form on basis of full-text screening).

**Authors, title, Year of publication, DOI/PMID, journal and other bibliographic data:**

No	Question	Options	Exclusion if	Additional notes
1	Is the full text available?	Yes (go to question 2)	No	Please try to get the full text via available university libraries, network of friends, addressing the authors directly
		No		
2	Is the full text in English, French, German or Polish?	Yes (go to question 3)	No	
		No		
3	Again: What type of source is the result?	Journal Paper (go to question 4)		The aim of this question is to verify type of source in case an abstract gave a false view
		Conference Paper (go to question 4)		
		Commentary	Commentary	
		Editorial	Editorial	
		Others (e.g. press article)	Others (e.g. press article)	
4	The article addresses, either in clinical setting or in animal models or in in vitro studies, the role of cathelicidin in:			
	HP	Yes		
	COPD	Yes		
	asthma	Yes		
	Exposure to organic dust	Yes		
		None of the above	No / None of 4a-d is confirmed	

Supplementary Material S4

Table S3. Characterization form on basis of full-text analysis.

No	Question	Option	Additional notes
1	The article comes from which domain?	clinical setting (including sample size and type of study)	
		animal model	
		in vitro study	
		review	
2	Goals of the article		
3	Description of the role/behaviour of cathelicidin in a selected lung disease or in exposure to organic dust provided by an article.		
4	Important results of an article		

Supplementary Material S5

Table S4. Data abstraction of study characteristics.

Study Ref.	Domain	Country/Region	Peptide	Clinical Setting Addressed	Sample Size	Important Result Abstract	PMID or DOI
[79]	in vitro study	Japan/Asia	LL-37	COPD	n.a.	LL-37 counteracted cigarette smoke extract-induced and prevented airway epithelial barrier dysfunction, including disruption of occludin and ZO-1. Use of LL-37 to counteract airway epithelial barrier dysfunction may have significant benefits for respiratory diseases such as asthma and COPD.	doi:10.1186/s12931-019-1226-4
[67]	animal model	Canada, USA/ N. America	CRAMP	asthma	n.a.	CRAMP decreased in lung tissue in murine model of asthma.	doi:10.3389/fimmu.2020.01932
25	review	Poland/Europe	LL-37	exposure to organic dust, HP, COPD, asthma	n.a.	LL-37 stimulates angiogenesis, induces proliferation of lung epithelial cells, accelerates wound closure of the airway epithelium, and provokes cytokine release (e.g., IL-8) and cell migration. LL-37 is also able to neutralize LPS, a heteropolymer produced by Gram-negative bacteria associated with organic dust, causative factor of HP, COPD, asthma.	PMID: 17655171
[55]	animal model	China/Asia	LL-37	exposure to organic dust, COPD	n.a.	The study evaluated the effect of cathelicidin LL-37 on corticosteroid insensitivity in COPD rat model, established by exposing rats to cigarette smoke and LPS. LL-37 enhanced the anti-inflammatory effect of budesonide in an additive manner. Treatment with combination of inhaled corticosteroids and LL-37 led to a significant increase in histone deacetylase-2 (HDAC2) expression and activity leading to restoring sensitivity to corticosteroid receptor alpha.	doi:10.1097/CM9.0000000000000107

[68]	in vitro study	UK/Europe	LL-37	COPD, asthma	n.a.	The study examined impact of citrullination on LL-37 direct antiviral activity against human rhinovirus (HRV), a common pathogen exacerbating asthma and COPD. Citrullination of LL-37 reduced its direct antiviral activity against HRV. Cathelicidin citrullination during infection may represent a novel viral evasion mechanism, likely applicable to a wide range of pathogens, and should therefore be considered in the design of therapeutic LL-37 derivatives.	doi:10.3389/fimmu. 2020.00085
[42]	clinical study (cohort study)	USA/N. America	LL-37	COPD	1609 individuals with COPD at 6, 12, 18 months	Assessment of the associations of plasma cathelicidin levels with cross-sectional and longitudinal COPD outcomes. Reduced cathelicidin plasma level was observed to be associated with lower lung function at baseline. No independent associations with longitudinal lung function decline or participant-reported COPD exacerbations were observed.	doi:10.15326/jcopdf.7.4.2020.0142
[39]	clinical study (cross-sectional study)	Poland/Europe	LL-37	COPD	55 individuals with COPD	Assessment of the levels of LL-37 in BALF and ELF in COPD and healthy controls was conducted. Changes in LL-37 levels in pulmonary compartment in COPD as such and in disease progression were observed. Namely, significantly elevated LL-37 in COPD GOLD I-II, while decreased levels of L-37 in COPD GOLD III-IV stage, compared to healthy individuals, were noted.	PMID: 23241112
[80]	in vitro study	The Netherlands/ Europe	LL-37	COPD	n.a.	The aim of the study was to investigate the combined effects of vitamin D (VD) and TGF-β1 on airway epithelial cell host defence in COPD. VD is an important regulator of respiratory host defence, e.g., by increasing LL-37 expression. TGF-β1 is increased in COPD. TGF-β1 was observed to reduce the VD-mediated LL-37 expression (i) directly by reducing the expression of an important transcription factor for LL-37 and (ii) indirectly via increases in CYP24A1 that promotes VD degradation.	doi:10.1159/000497415

[29]	review	USA/N. America	LL-37	asthma	n.a.	Emerging evidence indicates that vitamin D-mediated innate immunity, particularly through enhanced expression of the LL37, is important in host defences against respiratory tract pathogens. Observational studies suggest that vitamin D deficiency increases risk of respiratory infections.	doi:10.1007/s11882-009-0012-7
[40]	clinical study (cross-sectional study)	Turkey/Europe	LL-37	COPD	216 individuals (183 COPD, 33 healthy)	https://pubmed.ncbi.nlm.nih.gov/30774329/ accessed on 1. December 2021 The study investigated plasma LL-37 levels in patients with stable COPD. Plasma LL-37 levels were significantly lower in the COPD patients than in those of the control subjects. LL-37 levels were significantly lower in GOLD IV than in GOLD I, II, and III. Furthermore, in GOLD high-risk group, LL-37 and FEV1 were positively correlated. Study indicated that plasma LL-37 may play an important role in COPD. Decreased LL-37 levels may be particularly high risk for patients in COPD stage GOLD IV.	doi:10.2147/COPD.S185602
[58]	clinical study (cross-sectional study)	China/Asia	LL-37	COPD, asthma	352 individuals (62 healthy never smokers, 62 healthy smokers, 87 asthma, 73 COPD, 68 ACO)	The concentration of LL-37 in sputum from ACO (asthma–COPD overlap) patients was significantly elevated compared to asthma patients, whereas the levels decreased compared to those of COPD patients. Sputum LL-37 had a high AUC of the ROC curve in differentiating asthma and COPD. The sputum LL-37 level might be a biomarker for differentiating asthma and COPD.	doi:10.1038/s41598-019-55502-2

12	clinical study (cross-sectional study)	Poland/Europe	LL-37	COPD, exposure to organic dust	82 individuals (30 COPD, 36 healthy farmers, 16 healthy urban dwellers)	The study investigated levels of LL-37 in sputum of COPD and exposure to organic dust. Significantly higher LL-37 levels were found (i) in COPD compared to healthy individuals and (ii) in farmers compared to urban dwellers. Significantly higher LL-37 levels in sputum in COPD patients and farmers suggest the role of cathelicidin both in COPD and lung response to organic dust exposure.	PMID: 20047264
[52]	clinical study (cross-sectional study)	China/Asia	LL-37	COPD	36 individuals (18 COPD, 18 healthy)	The study investigated the effects of LL-37 on airway mucus overproduction in COPD. Overexpression of both LL-37 and MUC5AC mucin (a major mucin component of mucus) in airways of COPD patients as well as a correlation between them was observed. It was demonstrated that LL-37 induces MUC5AC mucin production by airway epithelial NCI-H292 cells. LL-37 enhances the mucus production in COPD airways, thus contributing to the progression of COPD.	doi:10.1016/j.bbrc.2013.11.074
[48]	in vitro study	The Netherlands/ Europe	LL-37	COPD, asthma	n.a.	Inflammatory lung diseases such as COPD and asthma are characterized by the increased presence of eosinophils and neutrophils. However, the mechanisms that mediate the influx of these cells are incompletely understood. The study showed that LL-37 chemoattracts both eosinophils and neutrophils. This activity is mediated via an FPR. Thus, LL-37 may play a role in inflammatory lung diseases such as COPD and asthma.	doi:10.1159/000092305

[53]	in vitro study	China/Asia	LL-37	COPD, asthma	n.a.	Enhanced mucus production contributes to COPD progress. The study investigated the mechanism for LL-37 inducing MUC5AC mucin production in airway epithelial cells. LL-37 induced TACE and EGFR activation as well as TGF-α and MUC5AC mucin production by NCI-H292 cells in a dose-dependent manner. LL-37 induces MUC5AC mucin production by airway epithelial cells via TACE-TGF-α-EGFR pathway and by that may contribute to COPD development.	doi:10.3109/01902148.2014.926434
[36]	animal model	Poland/Europe	CRAMP	HP, exposure to organic dust	n.a.	The usefulness of CRAMP in the treatment of pulmonary fibrosis was assessed in an HP murine model. CRAMP attenuated the immune reaction induced by mice chronic exposure to P. agglomerans and inhibited hydroxyproline and collagen deposition in the lung tissue of mice treated with bacteria extract. The beneficial effect of CRAMP on HP treatment was associated with restoring the balance in quantity of immune cells, cytokines production and synthesis of extracellular matrix components. The study suggests the usefulness of cathelicidin in preventing lung fibrosis; however, cathelicidin was not able to reverse pathological changes completely.	doi:10.1371/journal.pone.0251237
[37]	animal model	Poland/Europe	CRAMP	HP, exposure to organic dust	n.a.	The study investigated changes in CRAMP, laminin (LAM-A1), selected TLRs and chemokine levels in lung tissue in experimental HP in mice. CRAMP level declined in experimental HP. Significant alterations of CRAMP level in pulmonary compartment indicate the role of the cathelicidin in HP.	doi:10.1016/j.patbio.2015.03.002
[30]	review	Ireland/Europe	LL-37	asthma	n.a.	Vitamin D deficiency may impact the course of asthma due to decreasing the level of cathelicidin.	doi:10.1016/j.pupt.2015.02.004

[59]	clinical study (cohort study)	Turkey/Europe	LL-37	asthma	63 individuals (33 acute asthma/exacerbations, 30 stable asthma)	This study investigated the association of innate and adaptive immunity with acute asthma attacks by analysing the role of cathelicidin. Cathelicidin levels in serum were significantly higher in acute asthma/asthma exacerbations than in control group with stable asthma. Cathelicidin can be used to predict viral-induced acute asthma. It also may provide a potential new treatment target for acute asthma.	doi:10.1016/j.aller.2016.07.003
[33]	review	Australia/ Australia and Oceania	LL-37	COPD	n.a.	The paper discusses the two types of ALX/FPR2 receptor agonists: sustaining inflammation and resolving inflammation. LL-37 levels are elevated in sputum samples from stable COPD patients and are further increased during bacterial acute exacerbations of COPD. This normally protective molecule may contribute to persistent inflammation. Relative abundance and persistence of pro-inflammatory agonists such as LL-37 in COPD airways are likely to facilitate agonist-biased signalling that favours leukocyte recruitment, activation and survival. LL-37 is, according to the paper, a pro-inflammatory factor/mediator in the course of COPD.	doi:10.3978/j.issn.2072-1439.2014.08.08
[57]	clinical study (cross-sectional study)	China/Asia	LL-37	COPD, asthma	180 individuals (28 CF, 74 COPD, 34 asthma, 44 healthy)	The study investigated several components of innate immunity in induced sputum of patients with cystic fibrosis (CF), COPD, and asthma, and healthy control subjects. Cathelicidin levels were elevated in COPD patients compared to control subjects, while asthma patients had reduced cathelicidin levels, both compared to controls and COPD. Induced-sputum innate immune factor levels discriminate inflammatory changes in CF, COPD, and asthma, suggesting potential roles in pathophysiology as well as providing disease-specific biomarker patterns.	doi:10.1378/chest.128.4.2316

[43]	clinical study (cross-sectional study)	Australia/ Australia and Oceania	LL-37	COPD, asthma	165 individuals (44 COPD, 94 asthma, 27 healthy)	Antimicrobial proteins in sputum were significantly elevated in asthma and COPD compared with healthy controls. Antimicrobial proteins were positively correlated with airway neutrophils and negatively correlated with lung function and COPD symptoms. The level of LL-37 was significantly higher in COPD than in asthma. Activation of innate immune responses, including antimicrobial peptides such as cathelicidin, contributes to disease pathogenesis in COPD and asthma.	doi:10.1111/resp.12730
[63]	animal model	Hong Kong/Asia	CRAMP	asthma	n.a.	The role of antimicrobial peptide LL-37 in asthma exacerbation is unclear. Microbial infection, which is the most common inducer of asthma exacerbation, is accompanied by elevated LL-37. By using the ovalbumin-induced asthmatic model, intranasal administration of CRAMP in combination with ovalbumin during the allergen challenge stage significantly enhanced airway hyperresponsiveness and airway inflammation in sensitized mice, thereby implicating a deteriorating role of LL-37 in allergic asthma. This study shows that LL-37 triggers asthma exacerbation via the activation of eosinophils interacting with bronchial epithelial cells in inflammatory airways.	doi:10.1038/s41598-017-02085-5
[47]	clinical study (cohort study)	Norway/Europe	LL-37	COPD	356 individuals (COPD)	LL-37 levels were significantly higher in sputum of persons with COPD exacerbations compared to individuals with stable COPD.	doi:10.1371/journal.pone.0222449

[31]	review	USA/N. America	LL-37	asthma	n.a.	Epidemiological studies suggest that vitamin D deficiency predisposes to viral respiratory tract infections and mycobacterial infections and that vitamin D may play a role in the development and treatment of asthma. One of the main reasons for this is the role of vitamin D in stimulating the production of LL-37 in lung tissue.	doi:10.1016/B978-0-12-386960-9.00009-5
[60]	clinical study (cross-sectional study)	Turkey/Europe	LL-37	asthma	67 individuals (35 with asthma attacks, 32 stable asthma)	The mean cathelicidin serum level was significantly higher in the acute asthma group than in the controlled subjects with asthma. The mean serum vit D levels of the attack group were significantly lower than that of the controlled asthma group. Vit D deficiency showed a significant relationship in the development of asthma attacks independent of cathelicidin deficiency. Cathelicidin levels showed a significant positive association with asthma attacks and BMI.	doi:10.2500/aap.2015.36.3848
[61]	clinical study (cohort study)	UK/Europe	LL-37	asthma	25 individuals (10 asthma, 15 healthy)	The study investigated if neutrophil-related CXC chemokines and antimicrobial peptides are increased in rhinovirus-induced exacerbations of asthma. No significant differences were observed for LL-37 levels in BAL, neither between healthy controls and asthma patients, nor between asthma patients in exacerbation and in stable state. No significant differences were observed for LL-37 levels in BAL.	doi:10.1111/cea.12313
[81]	in vitro study	Belgium/Europe	LL-37	COPD	n.a.	The study analyses the direct role of vit D on cigarette smoke-exposed bronchial epithelial cells (BEC) from COPD patients and controls. Vit D significantly increased cathelicidin expression in BEC of both COPD subjects and controls. In conclusion, vit D supplementation may potentially reduce infectious exacerbations in COPD by the upregulation of cathelicidin in the bronchial epithelium.	doi:10.3390/nu11092138

[34]	review	UK/Europe	LL-37, CRAMP	COPD, asthma	n.a.	Both LL-37 and the murine cathelicidin mCRAMP have shown the ability to damage the viral envelope of a number of viruses, such as influenza A, HSV or dengue virus. Deficiency in LL-37 in humans leads to an increased susceptibility to infection. Exogenous LL-37, or vit D-mediated cathelicidin production, has potent direct antiviral activity against rhinovirus (RV). LL-37 has also shown the ability to modulate inflammation, which may prove to be a key property in the development of therapeutics that modulates the inflammatory response to viral infections.	doi:10.2217/fvl-2018-0016
[54]	clinical study (cross-sectional study)	UK/Europe	LL-37, CRAMP	COPD	56 individuals (37 COPD, 19 healthy)	Commonly prescribed therapy inhaled corticosteroids (ICS) accentuating pneumonia risk in COPD is poorly understood. ICS impairment of pulmonary immunity was dependent on suppression of cathelicidin, as ICS had no effect on bacterial loads in mice lacking cathelicidin (Camp-/-), and exogenous cathelicidin prevented ICS-mediated expansion of Streptococci within the microbiota and improved bacterial clearance. This suggests a central role for cathelicidin in suppression of anti-bacterial host defence by ICS in COPD. Therapeutic restoration of cathelicidin to boost antibacterial immunity and beneficially modulate the lung microbiota might be an effective strategy in COPD. Additionally, reduction of LL-37 was found in COPD GOLD 0–II patients versus healthy non-smokers. No significant differences were noted in regard in BALF.	doi:10.1126/scitranslmed.aav3879

[44]	animal model	Belgium/Europe	CRAMP	COPD	n.a.	COPD, which is characterized by an excessive inflammatory response of the airways, is often complicated by exacerbations, often caused by bacterial infections, e.g., by Haemophilus influenzae. The study revealed that faster and complete eradication of H. influenzae, in a murine model of COPD, was associated with upregulation of CRAMP mRNA during infection. Upregulation of CRAMP mRNA during H. influenza infection contributed to defence against the infection.	doi:10.1016/j.jsbmb.2018.10.021
[71]	in vitro study	Romania/Europe	LL-37	asthma	n.a.	Vitamin D increases the antiviral activity of bronchial epithelial cells in vitro. Exogenous vitamin D increased antiviral defences most likely via cathelicidin and innate interferon pathways. Cathelicidin had direct anti-rhinovirus activity.	doi:10.1016/j.antiviral.2016.11.004

[35]	clinical study (cross-sectional study)	China/Asia	LL-37	COPD, exposure to organic dust	60 subjects (28 COPD, 32 healthy)	The study evaluated role of LL-37 in COPD, both in a clinical setting and in in vitro experiments with lung epithelial cells exposed to LPS and cigarette smoke extraction (CSE). Increased LL-37 sputum levels in COPD patients were associated with airflow limitation. Elevated LL-37 levels in both airways and alveoli in COPD group were significantly increased compared with healthy controls. Through stimulation by CSE and LPS, the expression of LL-37 was increased in bronchial epithelial cells and alveolar epithelial cells. LL-37 promotes releasing inflammatory factor IL-8 and induces apoptosis of bronchial epithelial cells and alveolar epithelial cells. This study suggested that LL-37 may play an important role in the pathogenesis of COPD and may be a possible novel therapeutic target in COPD. Sputum: significantly increased LL-37 level in GOLD I–II and GOLD III–IV compared to healthy individuals. Significantly increased LL-37 in GOLD III–IV compared to GOLD I–II. Significantly increased in healthy smokers vs. healthy non-smokers. LL-37 levels in serum: no differences.	doi:10.1016/j.rmed.2012.08.018
[70]	clinical study (cohort study)	Mexico/N. America	LL-37	asthma	86 individuals	The study explored the effect of vitamin D supplementation on the colonization of pathogenic bacteria in the upper respiratory tract of asthma patients. Respiratory infections were drastically reduced, and this decrease was related to the number of patients who had high LL-37 levels in sputum. Treatment of asthma patients with vitamin D reduced respiratory infections, and this effect was related to the increase in cathelicidin LL-37.	doi:10.1016/j.cyto.2018.01.001

[32]	review	Hungary/Europe	LL-37	COPD, asthma	n.a.	This paper reviewed recent data on the role of vitamin D (VD) in the genesis of various immunological disorders. Vit D enhances the transcription of cathelicidin and, due to this, VD supplementation may be useful in the prevention or adjunct treatment of COPD and asthma.	doi:10.1586/ers.12.57
[62]	clinical study/cohort study	Germany/Europe	LL-37	asthma	42 individuals (19 asthma, 23 healthy controls)	The study investigated whether antimicrobial peptide levels in nasal secretions of asthma patients are lower than in healthy controls, and whether administration of the active form of vitamin D affects these antimicrobial peptide levels. No differences in LL-37 were detected between asthma patients and healthy controls. No significant differences in LL-37 levels were noted before and after vit D therapy. A short-term treatment with active vitamin D did not affect the level of LL-37.	doi:10.1371/journal.pone.0140986
[49]	clinical study/cohort study	The Netherlands/ Europe	LL-37	3COPD	36 individuals	The study investigated whether cigarette smoke CS induced damage-associated molecular pattern (DAMP) release or whether DAMP-mediated inflammation contributes to susceptibility for COPD. The relevant results of the study include: LL-37 increased CXCL8 secretion in AECs. LL-37 induced neutrophilic airway inflammation, exclusively in mice susceptible to CS-induced airway inflammation. LL-37 induced a proinflammatory response in airway epithelial cell AECs in COPD patients as well as in controls. LL-37 contributes to the development of COPD. LL-37 induces pronounced neutrophilic airway inflammation in mice susceptible to CS-induced airway neutrophilia.	doi:10.1152/ajplung.00135.2016

[82]	in vitro study	The Netherlands/ Europe	LL-37	asthma	n.a.	Asthma is accompanied by Th2-driven inflammation mediated by cytokines such as IL-4 and IL-13, and the effects of these cytokines on vitamin D metabolism and LL-37 expression are unknown. Therefore, the study investigated this with well-differentiated bronchial epithelial cells. The results showed that IL-13 enhances the ability of 25D3 to increase expression of LL-37. The results demonstrated that IL-13 induces vitamin D-dependent LL-37 expression, most likely by increasing CYP27B1.	doi:10.1128/IAI.06224-11
[51]	clinical study (cross-sectional study)	China/Asia	LL-37	COPD	60 individuals (20 healthy non-smokers without COPD, 22 smokers without COPD, 18 smokers with COPD)	The study examined the expression of LL-37 in small airways from smokers with COPD and controls, and then, the association between LL-37 expression in the epithelium and the structural changes of small airway remodelling was analysed. The results showed that LL-37 immunoreactivity in airway epithelium was significantly elevated in smokers with COPD compared with controls. In addition, the magnitude of LL-37 expression in epithelium was positively correlated with airway wall thickness and collagen deposition. In vitro, CSE-induced epithelial secretion of LL-37-promoted fibroblast collagen production. Finally, we showed that the formyl peptide receptor-like 1 (FPRL1)-dependent extracellular signal-regulated kinase (ERK) signalling pathway was essential for LL-37-induced collagen production in HFL-1 cells. These results suggest that after cigarette smoke exposure, the increased levels of LL-37 in airway epithelium could stimulate collagen production in the underlying lung fibroblasts and may contribute to small airway remodelling in COPD.	doi:10.1038/labinvest.2014.86

[41]	clinical study (cross-sectional study)	China/Asia	LL-37	COPD	135 individuals (84 COPD, 51 controls)	The study explored relationship between LL-37 plasma levels and exacerbation risk in patients with COPD. The plasma concentration of LL-37 was significantly lower in the high exacerbation risk group than in the control group. However, there was no significant difference between the low-risk group and high-risk group. Hospitalization frequency for COPD exacerbations was negatively correlated with plasma levels of LL-37. Low LL-37 plasma levels might predict exacerbation risk in COPD.	doi:10.3978/j.issn.2072-1439.2015.04.33
[69]	clinical study (cross-sectional study)	Sweden/Europe	LL-37	COPD	30 individuals (10 COPD GOLD III, 10 COPD GOLD IV, 10 healthy)	The study investigated impact of post-translation modification (citrullination, i.e., converting cationic peptidylarginine residues to neutral peptidyl citrulline) by peptidylarginine deiminases (PADIs) on LL-37 activity in COPD. PADI4, stored in granulocytes and extracellularly in the lumina of bronchi, was found in lung tissue of individuals suffering from COPD. In vitro, recombinant human PADI2 and PADI4 both caused a time- and dose-dependent LL-37 citrullination. The citrullination resulted in impaired antibacterial activity. Citrullinated LL-37 was less efficient at neutralizing LPS. Citrullinated LL-37 was more prone to degradation by proteases. The findings demonstrate that inflammation-dependent deiminases PADI2 and PADI4 can alter LL-37 activities, affecting the course of inflammatory disorders such as COPD.	doi:10.1165/rcmb.2010-0500OC

[50]	clinical study (cross-sectional study)	China/Asia	LL-37	COPD	29 individuals (10 healthy non-smokers, 9 smokers without COPD, 9 COPD smokers)	This study investigated if cathelicidin induces epithelial–mesenchymal transition (EMT) to promote airway remodelling in COPD. Significant EMT was found in the small airways of smokers both with and without COPD, as well as in the airways of COPD model mice. Downregulation of CRAMP in COPD mice, however, ameliorated airway EMT induced by cigarette smoke. Conversely, upregulation of CRAMP enhanced airway EMT in vivo. TACE, TGF-α, and EGFR were found to be involved in this process. In vitro, EMT induced by CSE and LL-37 was inhibited by blocking TACE, TGF-α, and EGFR expression. Cathelicidin promotes airway EMT by activating the TACE/TGF-α/EGFR signalling pathway. The LL-37 level was increased in lung tissue samples of COPD patients compared to healthy control.	doi:10.21037/atm-20-2196
[83]	clinical study (cross-sectional study)	USA/N. America	LL-37	asthma	16 individuals (asthma)	The objective of this study was to investigate the activation of vitamin D and antimicrobial peptides within the airways (BAL) during late-phase responses following allergen challenge of allergic subjects suffering from asthma. The LL-37 levels in unconcentrated BAL fluids were found significantly increased in allergen-challenged conditions compared to saline-challenged settings.	doi:10.1111/j.1365-2222.2011.03879.x

[66]	clinical study/ cohort study	Norway/Europe	LL-37	COPD	260 individuals (45 controls, 215 COPD)	We aimed to study AMP levels in stable COPD and during acute exacerbations of COPD (AECOPD) and to examine their relation to clinical parameters and inflammatory markers. Sputum AMP levels were higher in patients with stable COPD (n = 215) compared to controls (n = 45), and further changed during AECOPD (n = 56), with increased LL-37. In stable COPD, high sputum LL-37 levels were associated with increased risk of AECOPD, non-typeable H. influenzae colonisation, higher age, ex-smoking and higher levels of inflammatory markers. Altered levels of selected AMPs are linked to airway inflammation, infection and AECOPD, suggesting a role for these peptides in airway defence mechanisms in COPD.	doi:10.1183/13993003.01328-2016
[45]	clinical study/ cohort study	USA/N. America	LL-37	COPD	11 individuals	LL-37 levels were higher during exacerbation by non-typeable H. influenzae and M. catarrhalis. Significantly higher levels of LL-37 in COPD exacerbations due to bacterial infections indicate that LL-37 plays a role in COPD and exacerbates clinical conditions.	doi:10.1378/chest.10-2760
[56]	in vitro study	The Netherlands/Europe	LL-37	COPD	n.a.	The study explored if differential effects of the expression of immune defence genes is the reason behind the fact that COPD patients have increased risk of pneumonia when treated with fluticasone propionate (FP), whereas this is generally not the case with budesonide (BUD) treatment. No differences in LL-37 gene expression between treatment by BUD or FP were noted.	doi:10.1016/j.pupt.2018.04.002

[46]	clinical study/ cohort study	UK/Europe	LL-37	COPD	26 individuals (13 COPD, 13 controls)	The study investigated relationships between rhinovirus and bacterial infections and the role of antimicrobial peptides in COPD exacerbations. Induced sputum was collected before and repeatedly after rhinovirus infection, and virus and bacterial loads were measured. Significant correlation between viral load and LL-37 level and significant increases in LL-37 in sputum before and after the viral infection suggest LL-37 role in lung tissue response in COPD exacerbations. Sputum LL-37 levels were increased significantly from baseline in the COPD group and after infection, but there was no such change in controls.	doi:10.1164/rccm.201205-0806OC
[84]	animal model	China/Asia	CRAMP	asthma	n.a.	House dust mite extracts (HDM)-sensitized mice were immunized with a subcutaneous injection of HDM. These mice were then challenged with an intranasal administration of HDM. After the last challenge, mice were infected with an intranasal instillation with P. aeruginosa. Then, the score of tissue inflammation and CRAMP expression were measured in the lung. The effect of TGF-β1 on CRAMP and CYP27B1 in airway cells (16HBE) was analysed. TGF-β1 did not increase the levels of CRAMP in airway epithelial cells. Furthermore, vit Dis required for TGF β1-induced CRAMP in airway epithelial cells. CRAMP was significantly increased in TGF-β1/Vit D-treated 16HBE cells.	doi:10.18632/oncotarget.19826

[85]	clinical study (cross-sectional study)	USA/N. America	LL-37	asthma	205 individuals (102 controls (51 adults, 51 children), 103 asthma (50 adults, 53 children))	The study explored age-specific effects of vitamin D (VD) in asthma. A positive correlation between serum VD and LL-37 levels was found both in the paediatric and adult asthma groups. Based on these data, the authors speculate that VD supplementation in children with asthma could reduce atopy and boost protection from seasonal respiratory viruses due to increased serum LL-37 levels.	doi:10.1016/j.jaci.2012.01.044
[86]	animal model	China/Asia	CRAMP	asthma	n.a.	The study explored the impact of glucocorticoids on the innate immune system and antibacterial host defence in a murine asthma model. Inhaled budesonide enhanced lung infection in allergic mice exposed to P. aeruginosa and increased the number of viable bacteria in lung tissue. Budesonide decreased the expression of CRAMP, increased the number of internalized P. aeruginosa in OVA-challenged mice and in lung epithelial cell lines. These data indicate that inhaled budesonide can suppress pulmonary antibacterial host defence by downregulating CRAMP in a murine asthma model and in vitro.	doi:10.1186/1471-2172-14-7
[87]	in vitro study	Sweden/Europe	LL-37	COPD	n.a.	The study shows that osteopontin (OPN), a multifunctional glycoprotein that is highly upregulated in the airways of COPD patients, co-localizes with several antimicrobial proteins expressed in the airways. In vitro, OPN bound, among others, human beta defensin-3 (hBD-3) but showed low affinity for LL-37. Binding of OPN impaired the antibacterial activity against the important bacterial pathogens S. pneumoniae and P. aeruginosa. This suggests that osteopontin does not impair antibacterial activity of LL-37, but it impairs such activity of other antimicrobial peptides such as defensins.	doi:10.1371/journal.pone.0146192

[64]	in vitro study	UK/Europe	LL-37	COPD, asthma	n.a.	Vitamin D supplementation reduces risk of acute respiratory infections in vitamin D-deficient individuals, but the mechanisms by which such protection is mediated are incompletely understood. Thus, experiments were conducted to characterise the influence of vit D on responses of a respiratory epithelial cell line (A549 cells) to infection with a major group human rhinovirus (RV-16). These effects were associated with enhanced expression of the genes encoding the NF-κB inhibitor IκBα and the antimicrobial peptide cathelicidin LL-37.	doi:10.1016/j.jsbmb.2018.11.013
[88]	animal model	USA/N. America	CRAMP	asthma	n.a.	The study demonstrates that the interaction of CCR2 and Fpr2 with their endogenous ligands, including CRAMP, mediates the trafficking of dendritic cells, which are responsible for triggering adaptive immunity, within the inflamed lung.	doi:10.1074/jbc.M113.450635

Supplementary Material S6

Table S5. Changes in cathelicidin level in COPD and asthma reported by clinical studies included in the scoping review.

Cathelicidin Level *	Material	Control Group	Article
		**COPD**	
increased	lung tissue samples	healthy controls	Jiang et al., Ann. Transl. Med., 2021 [50]
increased	lung tissue samples	healthy controls	Sun et al., Lab. Invest., 2014 [51]
increased in GOLD I-II, decreased in GOLD III-IV	BALF/ELF	healthy controls	Golec et al., J. Biol. Regul. Homeost. Agents, 2012 [39]
decreased in sputum of GOLD 0-II/no differences in BALF	sputum, BALF	healthy smokers	Singanayagam et al., Sci. Transl. Med., 2019 [54]
increased	sputum	asthma patients	Huang et al., Sci. Rep., 2019 [58]
increased (GOLD I-II)	sputum	healthy controls	Golec et al., Ann. Agric. Environ. Med., 2009 [12]
increased	sputum	healthy controls	Xiao et al., Respir. Med., 2005 [57]
increased	sputum	asthma patients	Wright et al., Respirology, 2016 [43]
increased in exacerbations	sputum	stable COPD	Tangedal et al., PLoS One, 2019 [47]
increased	sputum	healthy controls	Zhang et al., Res. Commun., 2014 [52]
increased	sputum	healthy controls	Persson et al., Eur. Respir. J., 2017 [66]
increased after the viral infection/no change in controls	sputum	COPD before infection/healthy controls	Mallia et al., Am. J. Respir. Crit. Care Med., 2012 [46]
sputum: increased in GOLD I-II-III-IV/ increased in GOLD III-IV compared to GOLD I-II/serum: no differences	sputum, serum	healthy controls	Jiang et al., Respir. Med., 2012 [35]
decreased in COPD/lower in group IV than in groups I, II, and III	plasma	healthy controls	Uysal et al., Int. J. Chron. Obstruct. Pulmon. Dis., 2019 [40]
correlated with lung function decrease	plasma	correlated with lung function decrease	Burkes et al., Chronic Obstr. Pulm. Dis., 2020 [42]
decreased in COPD patients with high exacerbations risk/no difference in low risk	plasma	healthy controls	Yang et al., J. Thorac. Dis., 2015 [41]
		**Asthma**	
no significant difference	BALF	healthy controls	Rohde et al., Clin. Exp. Allergy, 2014 [61]
decreased in stable, mild asthma	BALF	following allergen challenge	Liu et al., Clin. Exp. Allergy, 2012 [83]
decreased	sputum	COPD patients	Huang et al., Sci. Rep., 2019 [58]
decreased	sputum	healthy controls, COPD patients	Xiao et al., Chest, 2005 [57]

decreased compared to COPD/increased compared to healthy controls	sputum	COPD patients, healthy controls	Wright et al., Respirology, 2016 [43]
decreased in stable asthma	serum	acute asthma/ exacerbations	Arikoglu et al., Allergol. Immunopathol. (Madr), 2017 [59]
decreased in stable asthma	serum	acute asthma/ exacerbations	Arikoglu et al., Allergy Asthma Proc., 2015 [60]
no significant difference	nasal swab	healthy controls	Thijs et al., PLoS One, 2015 [62]

* Only statistically significant differences were taken into consideration.
